# Oncogenic HPV Types Infection in Adolescents and University Women from North Portugal: From Self-Sampling to Cancer Prevention

**DOI:** 10.1155/2011/953469

**Published:** 2011-11-28

**Authors:** Jani Silva, Joana Ribeiro, Hugo Sousa, Fátima Cerqueira, Ana Luisa Teixeira, Ines Baldaque, Teresa Osório, Rui Medeiros

**Affiliations:** ^1^Molecular Oncology Group, Portuguese Institute of Oncology of Porto, Road Dr. António Bernardino Almeida, 4200-072 Porto, Portugal; ^2^CEBIMED, Faculty of Health Sciences, Fernando Pessoa University, 4200-150 Porto, Portugal; ^3^Molecular Virology Laboratory of Virology Service, Portuguese Institute of Oncology of Porto, Road Dr. António Bernardino Almeida, 4200-072 Porto, Portugal; ^4^ICBAS, Abel Salazar Institute for the Biomedical Sciences, University of Porto, 4099-003 Porto, Portugal; ^5^CEQUIMED, Faculty of Pharmacy, University of Porto, 4050-047 Porto, Portugal; ^6^LPCC, Liga Portuguesa Contra O Cancro, Nucleo Regional do Norte, 4200-177 Porto, Portugal

## Abstract

This study aimed to characterize the HPV infection status in adolescents and young university women in Portugal. The distribution of HPV genotypes was evaluated by PCR DNA genotyping after self-sampling collection from 435 women of exfoliated cervical cells using a commercial kit. We observed an overall frequency of HPV infection of 11.5%. Furthermore, HPV DNA prevalence was 16.6% in those young women that self-declared as sexually active. The more frequently detected HPV types were 31, 16, 53, and 61. Statistical analysis identified median age (OR = 3.56; *P* = 0.001), the number of lifetime sexual partners (OR = 4.50; *P* < 0.001), and years of sexual activity (OR = 2.36; *P* = 0.008) as risk factors for HPV acquisition. Hence, our study revealed that oncogenic HPV infection is common in young asymptomatic women Portuguese women, with a history of 2–5 sexual partners and over 2 year of sexual activity. Moreover, these results demonstrate that HPV detection performed in self-collected samples may be important to appraise better preventive strategies and to monitorize the influence of vaccination programmes within different populations.

## 1. Introduction

Genital Human papillomavirus (HPV) is highly prevalent in sexually active women. This infection has been established as the etiological agents of genital warts and squamous intraepithelial lesions of the cervix, and nevertheless, only certain types of HPVs are able to induce cervical cancer development [1–3].

Cervical cancer is one of the leading causes of mortality by cancer with approximately 495 000 women newly diagnosed each year [4]. In Portugal, cervical cancer is the 4th most frequent cancer with 950 new diagnosis and 378 deaths per year, and the second most frequent among women between the age of 15 and 44 [5].

Adolescents and young-adult women are more vulnerable to HPV infection. These observations are based on biological/physiological differences in the cervical epithelium. While in adults the predominant cell type is squamous, in adolescents the predominant cell type is columnar and metaplastic [6]. However most HPV infections spontaneously regress and only in a small percentage of cases the infection persists, low-grade intraepithelial lesions progress to high-grade lesions and, ultimately, develop into invasive cervical carcinoma [7]. It is now accepted that persistent infection by high-risk HPVs is a necessary but nonsufficient condition for the development of cervical cancer [8].

HPV molecular detection has been implemented in several countries and it was already proposed as the primary test for screening by replacing the Pap smear test [9–11]. Whereas HPV DNA tests have higher negative predictive value and sensitivity than conventional Pap smear but poor positive predictive value for identifying women with LSIL or cancer [9–11], they may be extremely useful in the identification of women with none or low risk of developing intraepithelial lesions [12].

Nevertheless, HPV genotyping may be considered a more important methodology than detection only, since it helps to identify the persistent infections which are predictive of HPV infection outcome (development of intraepithelial lesions or cervical cancer). Several *in-house* methodologies for HPV genotyping have been developed in hospitals and research centres, and they are financially advantageous to detect and characterize HPV in clinical specimens [13, 14].

Despite the incidence and mortality rates of cervical cancer in Portugal, until now no national screening program was implemented and there is no sufficient epidemiological data for HPV infection, particularly in young women. Screening programs in young women are essential to understand not only the epidemiology of HPV but also behaviours and community practices and consequently prevent the propagation of infection. Adolescents should be educated regarding HPV and associated risk factors of infection. Hence, they should also be encouraged to obtain suitable gynecological cares after initiating sexual activity [15, 16]. 

Several studies have reported lately that self-obtained samples from the anogenital tract were accurate and suitable for HPV-DNA testing and have shown a similar correlation to clinically obtained samples [17, 18]. Self-sampling does not require a vaginal speculum examination by clinicians, therefore it reduces the discomfort that turns screening unattractive even among women who have access to health cares. Besides, studies showed that HPV DNA test from self-collected samples has sensitivity equivalent or even superior to the cytological test [12, 17, 19]. 

The present study is the first to characterize the presence of HPV in adolescents and university women from northern region of Portugal using the self-sampling method. 

## 2. Subjects, Materials, and Methods

### 2.1. Study Population

 A population-based study was developed to obtain a profile of HPV infection status in the Northern region of Portugal. Between March and May 2010, female students aged from 14 to 30 years old, from eight high schools and two university institutions from northern region of Portugal, were invited to participate in the study. Ethics approval was obtained from the Ethics Committee of *University Fernando Pessoa* and all individuals included in the study gave their informed consent according to the Declaration of Helsinki, which in the case of women younger than 18 years was signed by parents/guardians, in accordance with the Portuguese legislation. 

This study was undertaken in 435 women (median age 17.0 ± 2.46 years old), who have answered an anonymous questionnaire for epidemiological characterization. The study population was characterized according to age, age of menarche (median age 12.0 ± 1.34), education level, HPV vaccination, sexual activity (*n* = 277, 63.7%), age at first sexual activity (16.0 ± 1.67), number of years after first sexual intercourse (2.0 ± 2.06), and lifetime number of partners (Table 1).

### 2.2. Specimen Collection

All participants were volunteers and informed of the study through workshops developed specifically to explain the study and self-sampling procedure. Briefly, exfoliated cervical cells were collected by self-sampling using the commercial kit *Universal Transport Media* (UTM) from Digene (Digene, Brasil), which contains a conical brush and a tube with transport media to stabilize the sample. The self-collection process consists to inserting a sterile brush into the vagina until their fingers reached their labia, and then rotating the brush five times in the same direction and remove which from the vagina and place which in UTM as suggested by the commercial kit instructions.

### 2.3. HPV Analysis and Genotyping

Sample processing included the concentration of cells by centrifugation, followed by DNA extraction using QiAmp DNA Blood mini Kit (QIAGEN, Hilden, Germany), according to manufacturer's instructions. DNA quality was assessed by measuring the absorbance at 260 nm using a UV/Visible spectrophotometer and its purity assessed by the ratio of the values of absorbance at 260/280 nm. 

To assess the validation of extraction method, the presence of genomic DNA was tested with a PCR protocol for amplification of beta-globin gene with the PCO_3_ primer 5′-ACA CAA CTG TGT TCA TAG C-3′ and BGII primer 5′-GTC TCC TTA AAC CTG TCT TG-3′.

The PCR reaction was performed in a 50 *μ*L solution with 1x Taq buffer, 4.0 mM MgCl_2_, 0.2 mM DNTP'S, 0.30 *μ*M each primer, 1 U de Taq DNA Polimerase, and 0.2 ug of genomic DNA. The amplification conditions were as follows: denaturation of DNA template at 95°C for 3 min, followed by 35 cycles of 94°C for 1 min, 55°C for 1 min, 72°C for 1 min, and a final extension step at 72°C for 10 min. The amplified fragment of 175 base pairs (bp) was analyzed by electrophoresis in 1.5% (w/v) agarose gels stained with ethidium bromide and visualized under UV light. 

HPV DNA was detected through PCR using two different pairs of consensus primers: GP5+/6+, which amplify a region of 150 bp [20]; and MY09/11 degenerated primers, which amplify a region of 449–458 bp depending on HPV type (as previously reported [1]). Both sets of primers amplify a highly conserved region of the HPV L1 gene and are potentially capable of detecting a large number of mucosal HPV types in a single PCR reaction [21].

PCR amplification reaction with GP5+/6+ primers was carried in a 50 *μ*L of reaction mixture with 1x PCR Buffer, 3.0 mM MgCl_2_, 0.2 mM DNTP'S, 0.30 *μ*M of each primer, 1 U of *Taq *DNA polymerase, and 0.2 ug of genomic DNA. Thermal cycling was performed as follows: initial denaturation of DNA template at 95°C for 4 min, followed by 40 cycles of 94°C for 30 s, 44°C for 60 sec, 72°C for 90 s, and a final extension step at 72°C for 10 min. The amplified fragment was analyzed by electrophoresis in 1.5% (w/v) agarose gels stained with ethidium bromide and visualized under UV light. 

PCR amplification reaction with MY09/11 was performed in a 50 *μ*L of reaction mixture with 1x PCR Buffer, 4.0 mM MgCl_2_, 0.2 mM DNTP'S, 0.40 *μ*M of each primer, 1 unit of *Taq *DNA polymerase, and 0.2 ug of genomic DNA. The amplification conditions include an initial denaturation at 95°C during 3 min, followed by 40 cycles of 94°C for 45 s, 55°C for 45 s, 72°C for 1 min, and a final extension step at 72°C for 5 min. The amplified fragment was analyzed by electrophoresis in 1.5% (w/v) agarose gels stained with ethidium bromide and visualized under UV light.

HPV-positive cases by amplification with primers MY09/11 were typed by RFLP analysis as described by Nobre et al. in 2008 [14]. Each restriction endonuclease reaction was performed in a 20 *μ*L final volume reaction, using 5 *μ*L of MY09/11 PCR product, 2 *μ*L of 10x recommended restriction buffer, and 10 units of each restriction endonucleases: PstI (New England BioLabs, R0140S), HaeIII (Fermentas Inc., #ER0151, Canada), DdeI (New England BioLabs, R0175L), and RsaI (New England BioLabs, R0167S). Digestion was performed at 37°C for 5 hours. The restricted fragments were separated by electrophoresis on 3% agarose gels with ethidium bromide staining and visualized under ultraviolet light. The identification of HPV types was executed using the algorithm proposed by Nobre et al. in 2008 [14].

The definition of HPV positivity was based on the result of MY09/11 and/or GP5+/6+ PCR amplification. Nonconcordant results by both primer sets were sent to sequentiate for confirmation of result. HPV genotyping was performed in MY09/11 positive cases and HPV genotypes were divided into four groups based on their oncogenic activity: high-risk types (HPV 16, 18, 31, 33, 35, 39, 45, 51, 52, 56, 58, and 59), probable high-risk types (26, 53, 66, 68, 73, 82), low-risk types (6, 11, 13, 40, 42, 43, 44, 54 and 55, 61, 70, 72, 81, and 89), and types of undetermined risk (30, 32, 34 and 64, 62, 67, 69, 71, 74, 83, 84, 85, 86, 87, 90, 91, 97, 102, 106) [22, 23].

### 2.4. Quality Criteria

To ensure the quality of the study, the following quality criteria were defined: (1) blind study; (2) inclusion of negative and positive controls in all PCR reactions: as negative control we have used double distilled water (dd H_2_O) replacing template DNA in the reaction mix; and as positive control we used an HPV positive sample used in the Virology Service for diagnosis of HPV infection using the commercial kit *hc2 High-Risk HPV DNA test* (QIAGEN, Hilden, Germany); (3) repetition of 100% of samples for validation of results; and (4) analysis of both PCR and RFLP results individually by three of the authors. Nonconcordant results were sent to sequentiate for confirmation of result.

### 2.5. Statistical Analysis 

Statistical Analysis was performed using the computer software *Statistical Package for Social Sciences* version 16.0 for Mac. Chi-square (*χ* ^2^) test was used to compare frequencies among groups. The OR (Odds Ratio) and its 95% Confidence interval were calculated as a measure of the association between the categorical variables (mean age, mean age of menarche, mean age of first sexual intercourse, educational level, and lifetime number of sexual partners) and HPV infection status.

## 3. Results

### 3.1. HPV Distribution

The presence of HPV was tested in 435 samples obtained by self-collection from young women and 50 positive cases were identified. Positive cases included one case from women who referred no initiation of sexual activity and 3 from which such information was not provided. For data analysis, we have considered only women who referred to have initiated sexual activity (*n* = 277), where HPV infection had a prevalence of 16.6% (46/277) (Table 2). 

HPV infection was analyzed according to age, age of first menarche, age of first sexual intercourse, number of years after first sexual intercourse, number of sexual partners, educational level, and HPV vaccination status (Table 2). When comparing the frequency of HPV regarding, the number of lifetime sexual partners, we observed increased frequency of HPV among those who had 2–5 partners (29.8%) and women who initiated sexual activity over two years have a higher frequency of HPV infection (24.0%). 

We observed that HPV was more frequent among university students (24.0%) compared with nonuniversity students (12.8%). However, within positive cases, the prevalence of high-risk HPV was higher for nonuniversity students in comparison to university students (47.9% versus 34.8%) (Table 2). Overall, 19 HPV different types were detected: 6 high risk (HPV16, 18, 31, 45, 56, and 58); 2 probable high risk (HPV53 and 66), 4 low risk (HPV6, 54, 61, and 89); and 7 types of unknown-risk (HPV30, 32, 71, 84, 86, 87, and 97) (Table 3). Notably, the most frequent types of HPV found in the study were the high-risk types, with HPV31 being the most frequent (15.2%) followed by the HPV16 (13.0%), HPV53, and HPV61 (8.7%).

### 3.2. Risk Estimation for Epidemiologic Characteristics

Statistical significant differences were found when comparing HPV distribution regarding education level, HPV vaccination, mean age, lifetime number of sexual partners, and number of years after first sexual intercourse (Table 4). We found a strong correlation between the presence of HPV infection and women aged over 17 years old (OR = 3.56; 95% CI 1.59–7.97; *P* = 0.001), women with 2–5 sexual partners (OR = 4.50, 95% IC 2.26–8.96, *P* < 0.001), and women who had initiated their sexual activity over 2 years (OR = 2.36, 95% IC 1.24–4.49, *P* = 0.008).

## 4. Discussion

HPV infection is one of the most common sexually transmitted infections and a major public health concern, especially during adolescence [24, 25]. Most HPV infections are asymptomatic and are efficiently controlled by the immune system, therefore, the outcome of HPV infection is variable, the infection is usually transient, and complete resolution is generally common within 12 to 24 months [26, 27]. However, persistent infection with one or more carcinogenic types of HPV may lead to the emergence of intraepithelial lesions, which may progress to high-grade dysplasia or in severe cases to invasive carcinoma [7]. 

Due to the worldwide distribution of HPV infection and to the high-incidence in undeveloped countries, many companies have attempted to developed low-cost tests with high efficiency to detect HPV. Self-sampling has been suggested as a useful tool for testing women for HPV infection mainly in low-resource populations or for people who have difficult access to health care [12]. Self-sampling has been referred to as having more than a few advantages compared with physician-collected samples for detection of HPV genital infection [17]. Self-sampling is a less costly and noninvasive method; it reduces the discomfort that some women associate to medical examination and the uneasiness that make screening programs less pleasant, even in women who have access to health care services [17, 28]. Several studies have shown that self-sampling has sensitivity for detecting lesions with low and high grade and even invasive cancer, which is equivalent or even superior to Pap smear [17, 18]. A recent meta-analysis revealed that self-sampling had a sensitivity ≥70% and a specificity of 88% compared to physician-sampling to detect the presence of HPV infection [29]. 

Although the number of samples was limited and further large-scale epidemiological studies are needed to assess the significance of less common genotypes, this study provides the first view on the spectrum of the most common mucosal HPV types in young Portuguese women. 

Epidemiologic studies refer to a prevalence of HPV infection from 2% to 44% in general population, with the highest prevalence in women aged 20 to 24 years old [30, 31]. Our results showed an overall frequency of 11.5% (50/435) positive cases for HPV, and considering sexual activity as an important discriminating factor for HPV acquisition, the frequency among young sexually active women was of 16.6% (46/277). This frequency is similar to those found in other studies, which refer that HPV infection among asymptomatic women may vary from 12 to 31.4% depending on the population [32–36]. 

According to several studies, the behavioral risk factors associated with the HPV infection in adolescents and young adult women include early age of first sexual intercourse [24, 37–39]. However, in our population sample, the age of first sexual intercourse did not seem to be a cofactor for HPV infection. Nevertheless, our results suggest that age is an important factor for HPV infection and it was more frequent in young women aged more than 17 years old than in younger women. Once more, these results are in agreement with several other studies referring to HPV infection as consistently more common in sexually active younger women [40].

The number of lifetime sexual partners is considered to be one of the major risk factors for HPV infection [39], and in our study, we observed that women with more than one sexual partner (2 to 5 partners) are associated with an increased probability of HPV infection. Furthermore, we observed increased probability of infection in women who had initiated their sexual activity over 2 years. 

Present results showed differences comparing HPV distribution regarding education level and HPV vaccination. Since the median age of first sexual intercourse was of 16.0 years old, it is expected that university students have more years of sexual life than nonuniversity students. Therefore, they would be exposed more frequently to a HPV infection than nonuniversity students. Regarding HPV vaccination, since the National program of Vaccination in Portugal started to include the HPV vaccine 2 years ago for girls between 13 and 18 years old, it is expected that these girls would be protected from acquiring an HPV infection. Therefore our data is correlated to these evidences and vaccination may reduce efficiently the infection by HPV. 

Despite these important epidemiological data, characterization of HPV genotype frequencies among populations is considered to be even more imperative. HPV diagnostic assays such as hybrid capture second-generation (HC2) and PCR-based methods employing the consensus primers, MY09/11 and GP5+/6+, allow the detection of a large number of HPV types, mainly high-risk types [28]. These primer sets have been widely used to study the natural history of HPVs and their role in the development of cervical cancer [28, 41].

In our study, we have used the genotyping method described by Nobre et al. in 2008, which allows a good discrimination of mucosal HPV types, including several HPV types that are underdiagnosed by current diagnostic methods. Our data showed that high-risk HPV types are the most frequent, with HPV31 and HPV16 having higher frequency. Surprisingly, HPV31 was found as the most frequent among our study. A recent meta-analysis shows that HPV31 may be the second or third most common type in European women with normal cytology [40, 42]. Despite HPV18 is a prevalent type worldwide and the second most common high-risk type associated with cervical cancer development, it was not among the more frequent in our study. However, HPV 18 infection leads to a rapid onset of cervical cancer [43], and it is expected to be more frequently found in high-grade lesions than in asymptomatic young women. Notably, 13 mucosal HPV types that are not detected by the majority of current commercial assays (HPV 34, 53, 61, 62, 66, 67, 70, 71, 73, 81, 83, 84, and 102) were detected in the present study.

Regarding our results, we must consider that after an open invitation to participate in the study, all participants were volunteers and informed of the study through workshops developed specifically to explain the study and self-sampling procedure. We cannot rule out the influence of two factors, that the attending students were more keen to know more about HPV and that subjects at higher risk of HPV infection are fear of being detected positive. However, we do think that the influence of those factors was reduced because knowing HPV results was optional, and only under the supervision of the gynecologist. Furthermore, our rate of participation was not conditioned by the cost of the test (which was free to all participants).

Molecular pathology methodologies have been demonstrated as important in the improvement of screening, diagnostic, or monitoring cancer, and genetic- or viral-associated diseases. Furthermore, they provide valuable tools for the new paradigm in Medicine known as personalized medicine [44–46]. The new molecular biology procedures use traditionally difficult material as in the case of formaldehyde fixation samples, and to analyse unstable molecules as in the case of RNA, let us consider new possibilities in the practical use of the new molecular pathology biomarkers. Therefore, DNA and RNA viruses are in the frontline of these possibilities due to their influencing of the development and evolution of some diseases [47, 48]. In the case of HPV, molecular epidemiology studies have been the key to the opening of a window to vaccination against cervical cancer. Several lines of evidence are demonstrating the putative role of HPV molecular biology detection in the screening of cervical cancer and its putative role in head and neck cancer [49–52].

It is acceptable to consider that, to achieve the maximum efficacy in health prevention strategies, we must maximize the knowledge of some populations about their epidemiology, costumes, and concerns and that is particularly important in those populations where no screening or vaccination has already been developed [53–56]. This data reinforces the urgency to perform genotyping studies to characterize the HPV genotype distribution worldwide, so that preventive and therapeutic strategies can be adapted to each population according to the HPV genotypes distribution [1, 14]. 

In conclusion, we have to refer that self-sampling may be a good alternative in substitution of the vaginal speculum examination by a clinician as a tool for primary screening of HPV infection in young asymptomatic women. This study contributes to a better understanding of the epidemiology of HPV infection among young Portuguese women. The exact knowledge of HPV profile in young women may be important to appraise the vaccination strategies within the different population.

## Figures and Tables

**Table 1:** Characteristics of the study participants.

Age and sexual intercourse	*n* (%)
Age (median ± sd^a^)	17.0 ± 2.46 (range 14 to 30)
Age of menarche (median ± sd^a^)	12.0 ± 1.34 (range 8 to 16)
Age of first sexual intercourse (median ± sd^a^)	16.0 ± 1.67 (range 13 to 22)
Number of years after first sexual intercourse (median ± sd^a^)	2.0 ± 2.06 (range 0 to 12)
Education level (*n* = 434)	
Elementary (≤9th grade)	27 (6.2)
High school (10th–12th grade)	296 (68.2)
University	111 (25.6)
HPV vaccination (*n* = 435)	
Yes	161 (37.0)
No	231 (53.1)
NR^b^	43 (9.9)
Sexual activity (*n* = 435)	
Yes	277 (63.7)
No	38 (8.7)
NR^b^	120 (27.6)
No. of Sexual partners (*n* = 277)	
1	174 (62.8)
2–5	94 (33.9)
NR^b^	9 (3.3)

^a^Standard deviation; ^b^Nonresponders.

**Table 2:** Distribution of HPV in sexually active women.

	HPV positive *n* (%)	**HPV HR^a^** *n* (%)	**HPV pHR^b^** *n* (%)	**HPV LR^c^** *n* (%)	**HPV UR^d^** *n* (%)	**INC^e^** *n* (%)
Total (*n* = 277)	46 (16.6)	**19 (41.3)**	**7 (15.2)**	**8 (17.4)**	**7 (15.2)**	**5 (10.9)**

Age (*n* = 277)						
≤17 (*n* = 107)	8 (7.5)	**6/8 (75.0)**	**—**	**—**	**1/8 (12.5)**	**1/8 (12.5)**
18–20 (*n* = 104)	19 (18.3)	**6/19 (31.6)**	**3/19 (15.8)**	**3/19 (15.8)**	**4/19 (21.0)**	**3/19 (15.8)**
>20 (*n* = 66)	19 (28.8)	**7/19 (36.9)**	**4/19 (21.1)**	**5/19 (26.3)**	**2/19 (10.5)**	**1/19 (5.3) **

≤17 (*n* = 107)	8 (7.5)	**6/8 (75.0)**	**—**	**—**	**1/8 (12.5)**	**1/8 (12.5)**
>17 (*n* = 161)	38 (22.4)	**13/38 (34.2)**	**7/38 (18.4)**	**8/38 (21.1)**	**6/38 (15.8)**	**4/38 (10.5) **

Age at first menarche (*n* = 275)						
≤12 (*n* = 151)	26 (17.2)	**9/26 (34.6)**	**5/26 (19.2)**	**4/26 (15.4)**	**5/26 (19.2)**	**3/26 (11.5)**
>12 (*n* = 124)	20 (16.1)	**10/20 (50.0)**	**2/20 (10.0)**	**4/20 (20.0)**	**2/20 (10.0)**	**2/20 (10.0) **

Age at first sexual intercourse (*n* = 271)						
≤16 (*n* = 143)	22 (15.4)	**10/22 (45.4)**	**4/22 (18.2)**	**2/22 (9.1)**	**4/22 (18.2)**	**2/22 (9.1)**
>16 (*n* = 128)	24 (18.8)	**9/24 (37.5)**	**3/24 (12.5)**	**6/24 (25.0)**	**3/24 (12.5)**	**3/24 (12.5) **

No. of Sexual partners (*n* = 268)						
1 (*n* = 174)	15 (8.6)	**8/15 (53.3)**	**1/15 (6.7)**	**2/15 (13.3)**	**3/15 (20.0)**	**1/15 (6.7)**
2–5 (*n* = 94)	28 (29.8)	**10/28 (35.7)**	**4/28 (14.3)**	**6/28 (21.4)**	**4/28 (14.3)**	**4/28 (14.3) **

No. of years after first sexual intercourse (*n* = 271)						
≤2 (*n* = 181)	23 (12.7)	**11/23 (47.9)**	**2/23 (8.7)**	**3/23 (13.0)**	**3/23 (13.0)**	**4/23 (17.4)**
>2 (*n* = 90)	23 (25.6)	**8/23 (34.8)**	**5/23 (21.7)**	**5/23 (21.7)**	**4/23 (17.4)**	**1/23 (4.3) **

Education level (*n* = 276)						
Nonuniversity (*n* = 180)	23 (12.8)	**11/23 (47.9)**	**4/23 (17.4)**	**1/23 (4.3)**	**2/23 (8.7)**	**5/23 (21.7)**
University (*n* = 96)	23 (24.0)	**8/23 (34.8)**	**3/23 (13.0)**	**7/23 (30.5)**	**5/23 (21.7)**	**—**

HPV vaccination (*n* = 251)						
No (*n* = 154)	34 (22.1)	**13/34 (38.2)**	**7/34 (20.6)**	**7/34 (20.6)**	**4/34 (11.8)**	**3/34 (8.8)**
Yes (*n* = 97)	9 (9.3)	**5/9 (55.6)**	**—**	**—**	**3/9 (33.3)**	**1/9 (11.1)**

^a^High-risk HPV; ^b^Probable high-risk HPV; ^c^Low-risk HPV; ^d^Undeterminated- risk HPV; ^e^Inconclusive. The frequencies represented in the bold values of the table refer only to HPV-positives cases.

**Table 3:** Distribution of different HPV types.

HPV positive cases (*n* = 46)	*n* (%)
HR HPV^a^ (*n* = 19)	
16	6 (13.0)
18	2 (4.3)
31	7 (15.2)
45	1 (2.2)
56	1 (2.2)
58	2 (4.3)
pHR HPV (*n* = 7)	
53	4 (8.7)
66	3 (6.5)
LR HPV^c^ (*n* = 8)	
6	1 (2.2)
54	2 (4.3)
61	4 (8.7)
89	1 (2.2)
UR HPV^d^ (*n* = 7)	
30	1 (2.2)
32	1 (2.2)
71	1 (2.2)
84	1 (2.2)
86	1 (2.2)
87	1 (2.2)
97	1 (2.2)

Nonidentified (*n* = 5)	

^a^High-risk HPV; ^b^Probable High-risk HPV; ^c^Low-risk HPV; ^d^Undeterminated- risk HPV.

**Table 4:** Odds Ratio (*OR*) analysis for HPV.

	*P*	OR^a^	95% CI^b^
Education level			
University versus nonuniversity	0.018	2.15	1.13–4.08

HPV vaccination			
Vaccinated versus nonvaccinated	0.009	0.36	0.16–0.79

Median age			
≤17 versus >17	**0.001**	**3.56**	**1.59–7.97**

Median age first menarche			
≤12 versus >12	0.810	0.92	0.49–1.75

Median age first sexual intercourse			
≤16 versus >16	0.461	1.27	0.67–2.40

No. of sexual partners			
1 versus 2–5	**<0.001**	**4.50**	**2.26–8.96**

No. of years after first sexual intercourse (*n* = 271)			
≤2 versus >2	**0.008**	**2.36**	**1.24–4.49**

^a^Odds Ratio; ^b^Confidence interval.
